# Free-hand Frameless Pinless Electromagnetic-Navigation (AXIEM™)-Guided Brain Lesion Biopsies: An Institution Based Experience from a Low-Middle-Income Country

**DOI:** 10.12669/pjms.40.12(PINS).11106

**Published:** 2024-12

**Authors:** Muhammad Aqeel Natt, Ahtesham Khizar, Haseeb Mehmood Qadri, Maryem Tanweer, Raahim Bashir, Abdur Rehman Baig, Muhammad Nauman Hasan, Asif Bashir

**Affiliations:** 1 Dr. Muhammad Aqeel Natt, MBBS, FCPS Assistant Professor Neurosurgery, Department of Neurosurgery Unit-I, Punjab Institute of Neurosciences, Lahore, Pakistan; 2 Dr. Ahtesham Khizar, MBBS, FCPS Senior Registrar Neurosurgery, Department of Neurosurgery Unit-I, Punjab Institute of Neurosciences, Lahore, Pakistan; 3 Dr. Haseeb Mehmood Qadri, MBBS Postgraduate Resident Neurosurgery, Department of Neurosurgery Unit-I, Punjab Institute of Neurosciences, Lahore, Pakistan; 4 Dr. Maryem Tanweer, MBBS Postgraduate Resident Neurosurgery, Department of Neurosurgery Unit-I, Punjab Institute of Neurosciences, Lahore, Pakistan; 5 Dr. Raahim Bashir, MBBS, Department of Neurosurgery Unit-I, Punjab Institute of Neurosciences, Lahore, Pakistan; 6 Dr. Abdul Rehman Baig, MBBS Postgraduate Resident Neurosurgery, Department of Neurosurgery Unit-I, Punjab Institute of Neurosciences, Lahore, Pakistan; 7 Dr. Muhammad Nauman Hasan, MBBS Postgraduate Resident Neurosurgery, Department of Neurosurgery Unit-I, Punjab Institute of Neurosciences, Lahore, Pakistan; 8 Dr. Asif Bashir, MD, FAANS, FACS Professor of Neurosurgery, Department of Neurosurgery Unit-I, Punjab Institute of Neurosciences, Lahore, Pakistan

**Keywords:** Electromagnetic field, Neuronavigation, Biopsy, Image-guided biopsy, Developing countries

## Abstract

**Objectives:**

To evaluate the precision and safety of a novel technique of free-hand frameless pinless AXIEM™-based navigation guided biopsy of deep-seated brain lesions in a low-middle income country.

**Methods:**

This retrospective study included 45 patients who underwent free-hand frameless pinless AXIEM™-based navigation guided biopsy of deep-seated brain lesions using the Medtronic-Stealth S7 system over a 5-year period (January 2019 to December 2023) at the Department of Neurosurgery, Punjab Institute of Neurosciences, Lahore, Pakistan.

**Results:**

A total of 45 patients were included in this study. There were 4 (8.9%) patients in the 1 to 20 years age group, 10 (22.2%) in 21 to 40 years, 24 (53.3%) in 41 to 60 years and 7 (15.6%) were above 60 years. Mean age (years) was 47.29 ± 17.192 SD. Mean time (minutes) for procedures was 37.87 ± 9.6 SD. There were 28 (62.2%) male and 17 (37.8%) female patients. Among patients reviewed in this study 2 (4.4%) had lesions in Corpus Callosum, 8 (17.8%) in Lobar region, 5 (11.1%) were Multiple/Metastatic and 30 (66.7%) were in Thalamus/Internal Capsule/Basal Ganglia. Size of lesions was less than 1 cm in 4 (8.9%) cases, 1-2 cm in 29 (64.4%) and 2 to 3 cm in 12 (26.7%) cases. There were 18 (40%) patients with lesions on the left side, 21 (46.7%) on the right side, 4 (8.9%) bilateral and 2 (4.4%) in the midline. Most cases were performed in local (40%) or local with IV sedation (48.9%) and a few in general anesthesia (11.1%). Biopsy results were successfully diagnostic in 40 (88.9%) and non-diagnostic in 5 (11.1%) patients. Glioblastoma WHO Grade IV was seen in 20 (44.4%) patients, Diffuse Astrocytoma WHO Grade II in 5 (11.1%), Anaplastic Astrocytoma WHO Grade III in 2 (4.4%), Pleomorphic Xanthoastrocytoma in 1 (2.2%), Lymphoma in 7 (15.6%) and Metastasis in 5 (11.1%) patients. Asymptomatic minor haemorrhage was seen in 3 (6.7%) patients, massive haemorrhage in 2 (4.4%), hydrocephalus in 1 (2.2%), surgical site infection in 1 (2.2%) and 38 (84.4%) patients had no complications.

**Conclusions:**

AXIEM™-based Medtronic-Stealth S7 is a quick, reliable, real-time and secure neuronavigation system for taking a free-hand, frameless and pinless biopsy of deep-seated lesions in the brain.

**List of Abbreviations:**
CT: Computed Tomography,
EM: Electromagnetic,
FBN: Frame-based navigation,
FLN: Frameless navigation,
GA: General anesthesia,
GBM: Glioblastoma multiforme,
IV: Intravenous,
LA: Local anesthesia,
MS: Multiple sclerosis,
PCNSL: Primary central nervous system lymphoma,
SB: Stereotactic biopsy,
SPSS: Statistical Package for Social Sciences,
WHO: World Health Organization.

## INTRODUCTION

The incidence of neoplasms of brain worldwide reported in one meta-analysis is 10.82 per 100,000 people, among them deep seated brain lesions are more challenging in terms of their difficult, nearly impossible surgical excision.^1^ Deep seated lesions pose diagnostic and management related challenge as surgical excision carries high morbidity and mortality but definitive histopathology is needed for neoadjuvant treatment. Patient’s comorbidities and anesthesia fitness problems further add on the misery. Deep seated pathologies, like primary central nervous system lymphoma (PCNSL), anaplastic astrocytoma, glioblastoma multiforme (GBM), tumefactive multiple sclerosis (MS) and infections are biopsied and confirmed by neuronavigation.^2^-^4^

Widely used standard methods for taking biopsy of brain tumors include: conventional free-hand brain biopsy, stereotactic brain biopsy and frameless neuronavigation guided biopsy.^1^,^5^ Free-hand biopsy is technically simple, can be done under local anesthesia (LA) or intravenous (IV) sedation but, being a blind procedure, not accurate and safe for deep lesions. Stereotactic biopsy (SB) is primarily indicated in deep seated intracranial pathologies, with frame on (frame-based neuronavigation, FBN) and without frame (frameless neuronavigation, FLN).^6^ Frame-based techniques have been considered the gold standard for many years, due to their superiority over free-hand biopsy, even then they are limited by many factors including need of general anesthesia (GA) most of the times, discomfort to the patient including pain because of the frame, difficulty to assemble the parts of the frame and to get a fresh radiological investigation.^5^

Although not new, neuronavigation is the focus of interest among Pakistani neurosurgeons. Neuronavigation ensures spatialisation between pre-operative image coordinates and patient’s anatomical coordinates.^1^ Latest neuronavigation systems ensure real-time intraoperative navigation making use of radiology, accurate specification and localization of deep seated as well as cortical lesions, and multiplanar image reconstruction.^7^ Frameless neuronavigation has two modes of navigation either through Optical tracking system or AXIEM™-based system. Optical system needs head fixation with a Mayfield clamp so further adds misery of fixing a hardware on head just like frame and usually needs GA. AXIEM™-based system, on the other hand, avoids this hazard and allows head movements without compromising accuracy.

Literature is scanty of any technique of getting biopsy of deep seated lesions in surgically unfit patients. Any technique that is technically simple, less time consuming and can be done without GA will help these patients in further treatment. We have combined the simplicity of free-hand biopsy with the accuracy of the AXIEM™-based system. This hybridisation has evolved in a novel technique of free-hand frameless pinless AXIEM™-based neuronavigation biopsy.

This retrospective study examines accuracy and safety of this AXIEM™-based free-hand frameless biopsy technique in the management of deep brain space-occupying lesions (SOLs) which are not candidates for surgical excision either due to higher chances of complications or fitness issues. This employs an AXIEM™-based neuronavigation system from Medtronics Stealthstation S7, which couples a typical side-cutting biopsy cannula with an Electromagnetic (EM) stylet for navigating the entry site, trajectory and target site, allowing tip tracking without the use of a frame. To the best of our extensive search using PubMed, Google Scholar and Scopus, this is the first study from Pakistan analyzing frameless neuronavigation.

## METHODS

A retrospective observational study was conducted at the Department of Neurosurgery, Punjab Institute of Neurosciences, Lahore, Pakistan over a period of five years (January 2019 to December 2023). This was a non-probability based consecutive case series of 45 patients with different age groups.

### Ethical Approval:

This study was authorized by our institution’s ethical review board with reference no. 1753/IRB/PINS/APPROVAL/2024, dated March 6, 2024. In our center, it is a standard procedure to get written informed consent from every participant before utilizing their data for medical research. Their files were reviewed and data was collected in a preformed Google form.

### Inclusion Criteria:

• Patients with lesions less than 3 cm in size.
• Lesions located in corpus callosum, lobar region, thalamus, Internal Capsule and Basal Ganglia.
• Patients with multiple lesions/metastasis.
• Older patients with comorbidities and unsuitable for GA.

### Exclusion Criteria:

• Lesions more than 3 cm in size.
• Lesions in brainstem region.
• Biopsies taken by other stereotactic techniques.

### Data Collection Procedure:

This study included 45 patients, meeting the inclusion and exclusion criterias, with brain lesions who underwent AXIEM™-based navigation guided biopsy at the Department of Neurosurgery Unit-III, Punjab Institute of Neurosciences, Lahore, Pakistan. Data collected includes the patient’s age, gender, location of lesion, size of lesion, type of anesthesia given, biopsy results and complications. For free-hand frameless pinless biopsies, the AXIEM™-based Medtronic-Stealth S7 system was used without any rigid head frame or clamp. Guidance mode of Stealth S7 was used for accurate positioning of the target site and coupling standard side cutting biopsy cannula with EM-stylet was used for tracking of tip entry site, trajectory and target site. Majority of cases were performed under LA, LA with IV sedation and a few requiring GA. Postoperatively patients were evaluated by using a non-contrast computed tomography (CT) brain for any immediate complications and histopathology was used to check accuracy of biopsy samples.

### Data Analysis Procedure:

Data was analyzed by using Statistical Package for Social Sciences (SPSS) version 26. For stratification purposes age groups were compared with gender, location of lesion, size of lesion, type of anesthesia given, biopsy results and complications. P-values were calculated taking < 0.05 as significant.

## RESULTS

A total of 45 patients were included in this study belonging to different age groups; 1-20 years, 21 to 40 years, 41 to 60 years and above 60 years as shown in Table-I. In one to twenty years 4 (8.9%), 10 (22.2%) in 21 to 40 years, 24 (53.3%) in 41 to 60 years and 7 (15.6%) were above 60 years. Mean age was 47.29 ± 17.192 SD with minimum age of 12 years and maximum of 72 years. Mean time for procedures was 37.87 ± 9.6 SD with minimum of 15 minutes and maximum of 56 minutes. There were 28 (62.2%) male and 17 (37.8%) female patients. Among patients reviewed in this study 2 (4.4%) had lesions in Corpus Callosum, 8 (17.8%) in the Lobar region, 5 (11.1%) were Multiple/Metastatic and 30 (66.7%) were in Thalamus/Internal Capsule/Basal Ganglia. Size of lesions was categorized as less than 1 cm, 1 to 2 cm and 2 to 3 cm. 4 (8.9%) were less than 1 cm, 29 (64.4%) were 1 to 2 cm and 12 (26.7%) were 2 to 3 cm. There were 18 (40%) patients with lesions on the left side, 21 (46.7%) on the right side, 4 (8.9%) bilateral and 2 (4.4%) in the midline. Most cases were performed in LA (40%) or LA with IV sedation (48.9%) and a few in GA (11.1%). Biopsy results were successfully diagnostic in 40 (88.9%) and non-diagnostic in 5 (11.1%) patients. Glioblastoma WHO Grade IV was seen in 20 (44.4%) patients, Diffuse Astrocytoma WHO Grade II in 5 (11.1%), Anaplastic Astrocytoma WHO Grade III in 2 (4.4%), Pleomorphic Xanthoastrocytoma in 1 (2.2%), Lymphoma in 7 (15.6%) and Metastasis in 5 (11.1%). Asymptomatic minor hemorrhage was seen in 3 (6.7%) patients, massive hemorrhage in 2 (4.4%), hydrocephalus in 1 (2.2%), surgical site infection in 1 (2.2%) and 38 (84.4%) patients had no complications.

**Table-I:** Different parameters involved in the study.

Parameters		No. of patients (Total: 45)	Percentage (%)
Age group	1 to 20 years	4	8.9
	21 to 40 years	10	22.2
	41 to 60 years	24	53.3
	Above 60 years	7	15.6
Mean age (years)		47.29 ± 17.192 SD	
Mean time of procedure (minutes)		37.87 ± 9.6 SD	
Gender	Male	28	62.2
	Female	17	37.8
Location of lesion	Corpus Callosum	2	4.4
	Lobar	8	17.8
	Multiple/Mets	5	11.1
	Thalamus/Internal Capsule/Basal Ganglia	30	66.7
Size of lesion	Less than 1 cm	4	8.9
	1 to 2 cm	29	64.4
	2 to 3 cm	12	26.7
Laterality of lesion	Left	18	40
	Right	21	46.7
	Bilateral	4	8.9
	Midline	2	4.4
Type of anesthesia given	General	5	11.1
	Local	18	40
	Local with IV sedation	22	48.9
Biopsy results	Diagnostic	40	88.9
	Non-diagnostic	5	11.1
Histopathology	Glioblastoma WHO Grade IV	20	44.4
	Diffuse Astrocytoma WHO Grade II	5	11.1
	Anaplastic Astrocytoma WHO Grade III	2	4.4
	Pleomorphic Xanthoastrocytoma	1	2.2
	Lymphoma	7	15.6
	Metastasis	5	11.1
	Inconclusive	5	11.1
Complications	Asymptomatic minor hemorrhage	3	6.7
	Massive hemorrhage	2	4.4
	Hydrocephalus	1	2.2
	Surgical site infection	1	2.2
	None	38	84.4

### Post-stratification results:

### Gender distribution:

Gender distribution in different age groups in the study is shown in Table-II. Male were 3 (10.7%) and 1 (5.9%) female belonged to 1 to 20 years. Male were 7 (25%) and 3 (17.7%) female patients were in the 21 to 40 years category. Male were 15 (53.6%) and 9 (52.9%) female patients were in the 41 to 60 years age group and 3 (10.7%) male and 4 (23.5%) female were above 60 years of age with the P-value of 0.647.

**Table-II:** Gender distribution in different age groups.

Age group	Gender			

	Male	Female	Total	P-value
1 to 20 years	3 (10.7%)	1 (5.9%)	4 (8.9%)	0.647
21 to 40 years	7 (25%)	3 (17.7%)	10 (22.2%)	
41 to 60 years	15 (53.6%)	9 (52.9%)	24 (53.3%)	
Above 60 years	3 (10.7%)	4 (23.5%)	7 (15.6%)	
Total	28 (100%)	17 (100%)	45 (100%)	

### Location of lesions:

Location of lesions in different age groups is given in Table. III. 1 (50%) Corpus Callosum lesion was in 21 to 40 years and another 1 (50%) in 41 to 60 years. 3 (37.5%) lobar lesions belonged to 21 to 40 years, 4 (50%) to 41 to 60 years and only 1 (12.5%) above 60 years. 3 (60%) Multiple/Mets were in 41 to 60 years and 2 (40%) in above 60 years. 4 (8.9%) Thalamus/ Internal Capsule/ Basal Ganglia lesions were in the 1 to 20 years age group, 10 (22.2%) in 21 to 40 years, 24 (53.3%) in 41 to 60 years and 7 (15.6%) were in above 60 years with the P-value of 0.622.

**Table-III:** Location of lesion in different age groups.

Age group	Location of lesion					

	Corpus Callosum	Lobar	Multiple/Mets	Thalamus/Internal Capsule/ Basal Ganglia	Total	P-value
1 to 20 years	0 (0%)	0 (0%)	0 (0%)	4 (14.8%)	4 (8.9%)	0.622
21 to 40 years	1 (50%)	3 (37.5%)	0 (0%)	6 (22.2%)	10 (22.2%)	
41 to 60 years	1 (50%)	4 (50%)	3 (60%)	16 (59.2%)	24 (53.3%)	
Above 60 years	0 (0%)	1 (12.5%)	2 (40%)	4 (14.8%)	7 (15.6%)	
Total	2 (100%)	8 (100%)	5 (100%)	27 (100%)	45 (100%)	

### Size of lesions:

About 2 (50%) lesions were less than 1 cm in size in 1 to 20 years of age group, 1 (25%) in 21 to 40 years and 1 (25%) above 60 years as shown in Table-IV. For 1 to 2 cm lesions, 1 (3.5%) in 1 to 20 years, 9 (31%) in 21 to 40 years, 17 (58.6%) in 41 to 60 years and 2 (6.9%) above 60 years. Moreover, for 2 to 3 cm sized lesions, 1 (8.3%) belonged to 1 to 20 years, 7 (58.3%) to 41 to 60 years and 4 (33.3%) were above 60 years with a P-value of 0.005.

**Table-IV:** Size of lesion in different age groups.

Age group	Size of lesion				

	Less than 1 cm	1 to 2 cm	2 to 3 cm	Total	P-value
1 to 20 years	2 (50%)	1 (3.5%)	1 (8.3%)	4 (8.9%)	0.005
21 to 40 years	1 (25%)	9 (31%)	0 (0%)	10 (22.2%)	
41 to 60 years	0 (0%)	17 (58.6%)	7 (58.3%)	24 (53.3%)	
Above 60 years	1 (25%)	2 (6.9%)	4 (33.3%)	7 (15.6%)	
Total	4 (100%)	29 (100%)	12 (100%)	45 (100%)	

### Laterality of lesions:

Lesions were on the left side in 1 (5.6%), 4 (22.2%), 11 (61.1%) and 2 (11.1%) patients in 1 to 20 years, 21 to 40 years, 41 to 60 and above 60 years age groups. Lesions on the right side were in 3 (14.3%), 5 (23.8%), 9 (42.9%) and 4 (19%) in 1 to 20 years, 21 to 40 years, 41 to 60 years and above 60 years. Patients with bilateral lesions were 3 (75%) and 1 (25%) in 41 to 60 years and above 60 years age groups. Midline lesions were 1 (50%) in 21 to years and 1 (50%) in 41 to 60 years of age groups with the P-value of 0.836. (Table-V)

**Table-V:** Laterality of lesion in different age groups.

Age group	Laterality of lesion					

	Left	Right	Bilateral	Midline	Total	P-value
1 to 20 years	1 (5.6%)	3 (14.3%)	0 (0%)	0 (0%)	4 (8.9%)	0.836
21 to 40 years	4 (22.2%)	5 (23.8%)	0 (0%)	1 (50%)	10 (22.2%)	
41 to 60 years	11 (61.1%)	9 (42.9%)	3 (75%)	1 (50%)	24 (53.3%)	
Above 60 years	2 (11.1)	4 (19%)	1 (25%)	0 (0%)	7 (15.6%)	
Total	18 (100%)	21 (100%)	4 (100%)	2 (100%)	45 (100%)	

### Type of anesthesia given:

4 (80%) cases had GA in the 1 to 20 years age group and only 1 (20%) had GA above 60 years. 5 (27.3%) cases in the 21 to 40 years age group, 9 (50%) in 41 to 60 years and 4 (22.2%) above 60 years of age were purely done in LA. 5 (22.7%), 14 (63.6%) and 3 (13.6%) cases were performed in LA with IV sedation belonging to 21 to 40 years, 41 to 60 years and above 60 years age group, respectively with the P-value of < 0.001. (Table-VI)

**Table-VI:** Type of anesthesia given in different age groups.

Age group	Type of anesthesia given				

	General	Local	Local with IV sedation	Total	P-value
1 to 20 years	4 (80%)	0 (0%)	0 (0%)	4 (8.9%)	< 0.001
21 to 40 years	0 (0%)	5 (27.8%)	5 (22.7%)	10 (22.2%)	
41 to 60 years	1 (20%)	9 (50%)	14 (63.6%)	24 (53.3%)	
Above 60 years	0 (0%)	4 (22.2%)	3 (13.6%)	7 (15.6%)	
Total	5 (100%)	18 (100%)	22 (100%)	45 (100%)	

### Biopsy results:

In 1 to 20 years 3 (7.5%), 8 (20%), 23 (57.5%) and 6 (15%) biopsies were diagnostic, 21 to 40 years, 41 to 60 years and above 60 years of age group. 1 (20%) was non-diagnostic in 1 to 20 years, 2 (40%) were not diagnostic in 21 to 40 years age group, 1 (20%) in 41 to 60 years and 1 (20%) was non-diagnostic above 60 years with the P-value of 0.419 as given in Table-VII.

**Table-VII:** Biopsy results in different age groups.

Age group	Biopsy results			

	Diagnostic	Non-diagnostic	Total	P-value
1 to 20 years	3 (7.5%)	1 (20%)	4 (8.9%)	0.419
21 to 40 years	8 (20%)	2 (40%)	10 (22.2%)	
41 to 60 years	23 (57.5%)	1 (20%)	24 (53.3%)	
Above 60 years	6 (15%)	1 (20%)	7 (15.6%)	
Total	40 (100%)	5 (100%)	45 (100%)	

### Histopathology:

**Fig.1:**
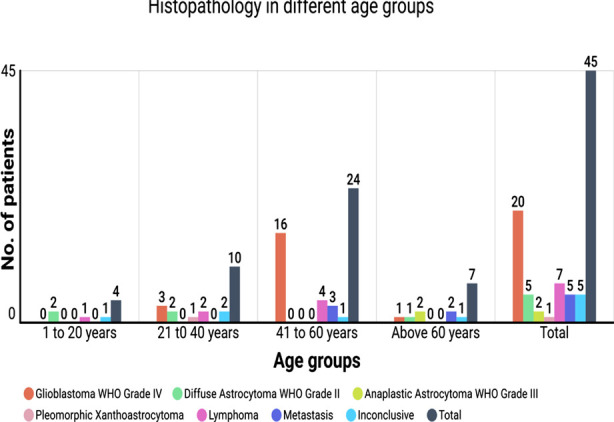
Histopathology in different age groups.

Histopathology revealed Glioblastoma WHO Grade IV in 3 (15%), 16 (80%) and 1 (5%) patients in 21 to 40 years, 41 to 60 years and above 60 years. Diffuse Astrocytoma WHO Grade II was seen in 2 (40%), 2 (40%) and 1 (20%) patient in 1 to 20 years, 21 to 40 years and above 60 years. Anaplastic Astrocytoma WHO Grade-III was seen in 2 (100%) patients of above 60 years age group. Pleomorphic Xanthoastrocytoma was seen in only 1 (100%) case in the 21 to 40 years age group. Lymphoma was seen in 1 (14.3%), 2 (28.6%) and 4 (57.1%) cases in 1 to 20 years, 21 to 40 years and 41 to 60 years. Metastasis was present in 3 (60%) and 2 (40%) patients in 41 to 60 years and above 60 years, respectively with the P-value of 0.005. (Fig.1) Moreover, there were 1 (20%), 2 (40%), 1 (20%) and 1 (20%) case in 1 to 20 years, 21 to 40 years, 41 to 60 years and above 60 years which had inconclusive histopathology.

### Complications:

Complications in different age groups are mentioned in Table. VIII. Asymptomatic minor hemorrhage occurred in 1 (33.3%) patient in 21 to 40 years, 1 (33.3%) in 41 to 60 years and 1 (33.3%) above 60 years. Massive hemorrhage was seen in 1 (50%) case in 41 to 60 years and another 1 (50%) above 60 years of age. Hydrocephalus was only seen in 1 case which was above 60 years of age. Surgical site infection was also seen in only 1 case belonging to 21 to 40 years of age group. 4 (10.5%) in 1 to 20 years, 8 (21%) in 21 to 40 years, 22 (57.9%) in 41 to 60 years and 4 (10.5%) above 60 years of age group had no complications with the P-value of 0.351. Asymptomatic hemorrhage was treated conservatively; large hemorrhage necessitated evacuation, a shunting procedure for hydrocephalus, and antibiotics for infection.

**Table-VIII:** Complications in different age groups.

Age group	Complications						

	Asymptomatic minor hemorrhage	Massive hemorrhage	Hydrocephalus	Surgical site infection	None	Total	P-value
1 to 20 years	0 (0%)	0 (0%)	0 (0%)	0 (0%)	4 (10.5%)	4 (8.9%)	0.351
21 to 40 years	1 (33.3%)	0 (0%)	0 (0%)	1 (100%)	8 (21%)	10 (22.2%)	
41 to 60 years	1 (33.3%)	1 (50%)	0 (0%)	0 (0%)	22 (57.9%)	24 (53.3%)	
Above 60 years	1 (33.3%)	1 (50%)	1 (100%)	0 (0%)	4 (10.5%)	7 (15.6%)	
Total	3 (100%)	2 (100%)	1 (100%)	1 (100%)	38 (100%)	45 (100%)	

## DISCUSSION

Planning for a safe puncture route, an appropriate puncture based on the preoperative plan, and needle stability for handling related to tissue aspiration are all important elements in brain tumor biopsy. Aside from traditional free-hand biopsy, there are three basic types of brain biopsy systems available today: frame-based stereotactic systems, frameless arm-based stereotactic systems, and frameless skull-mounted systems.^1^,^5^ All types of techniques or systems have some limitations in the form of accuracy, difficulty for patient and surgeon to handle rigid head frame and take images, need for head fixation, cumbersome instrument setup, and anesthesia requirement.^8^ Considering the patients with deep seated lesions who are already jeopardized by disease and have high risks of complications, we are limited to offer surgery/biopsy in either way. We need to have a simple, safe, accurate and precise solution. To overcome the above problems people are using frameless neuronavigation systems both optical or electromagnetic systems. Procedure can be done in LA or GA. Rigid head fixation is required to eliminate the danger of discrepancy between the head and the holding arm caused by patient movements during the operation, regardless of the anesthetic type used. We employ AXIEM™-based Electromagnetic (EM)-stylet Guidance Mode in our department to navigate and monitor ventricular catheter tips in slit ventricles and complex hydrocephalus. We can validate the entrance and target location, as well as follow the trajectory to avoid any vascular or eloquent structures, during preoperative planning. In order to utilize this concept for lesion biopsies, we came up with a novel technique that couples an EM-stylet with a biopsy needle side by side and uses Guidance Mode to do real-time navigation safely and precisely throughout the procedure. Only LA is required at the location of the burr hole, and the remainder of the operation is completed while the patient is awake and without the need of any other devices. Few individuals require IV sedation or GA. We can collect tissue samples with our free hands in numerous directions and at varying depths.

There is a lack of strong evidence preferring frame-based stereotactic biopsies over frameless neuronavigation and vice versa in the existing English scientific literature.^4^,^9^ Frame-based neuronavigation (FBN) and frameless neuronavigation (FLN) techniques have no paramount clinical differences in terms of diagnostic efficacy, patient morbidity and mortality. Although used more than FLN, the utility of FBN is under question these days due to patient’s trouble, complexity of assembling and maintaining frame and the cumulative duration of imaging and procedural time.^4^,^6^,^9^ We made use of a novel technique of free-hand FLN guided biopsy among 45 patients at our center, owing to the comparable efficacy of FLN with FBN, and simultaneous avoidance of cons of FLN.

Out of the 45 patients biopsied during the past four years, 62.2% were male similar to a ten-year retrospective study conducted by Dutch clinicians who biopsied 57.66% male cases. Taweesomboonyat et al.^3^ also had a 54.1% majority of male cases while performing neuronavigation-guided biopsies under GA. These findings are consistent with the literature review by M. Hillembrand and his colleagues who reviewed 13 studies to conclude that deep seated cerebral lesions are more common among males.^10^,^11^ The mean age of included patients in our study was 47.29 ± 17.192 years, which is a slightly younger age of presentation than that reported in the recent scientific literature, 51.5 ± 8.6 years attributing the early incidence of deep seated intracranial lesions in Pakistani population.^4^

**Fig.2:**
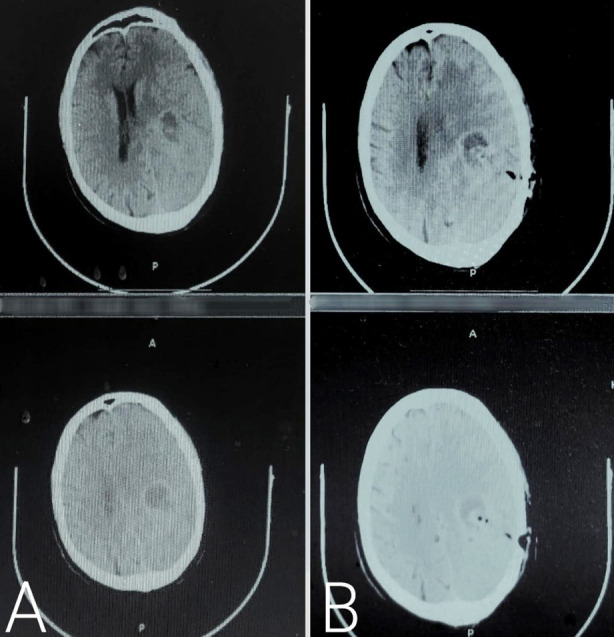
Per-operative imaging on StealthStation S7 **A:** Before biopsy, **B:** After biopsy.

About 64.4% lesions biopsied ranged in size from 1-2 cm, owing to our inclusion criterion of dealing lesions less than 3 cm in size. Existing retrospective studies from Nepal and Thailand document the mean size of biopsied lesions 3.34 ± 0.92 cm and 3.80 ± 2.1 cm, without taking into account size-specific criteria.^3^,^6^ An example of per-operative images on StealthStation S7 before and after biopsy are shown in Fig.2.

Lobar lesions accounted for 17.8% of the total cases, while deep cortical and multiple/metastatic lesions biopsies were taken in 82.2% patients. This contrasts with findings of Lobão et al.^12^ and Bishokarma et al.^6^ who operated 68.8% and 52.17% lobar lesions, respectively. Majority lesions in our study were located in the right cerebral hemisphere, i.e. in 46.7% cases, which is a comparable finding (56.52%) to a Nepalese study, yet contrasting from a Thai study in which 50.6% lesions were left-hemispheric.^3^,^6^

**Fig.3:**
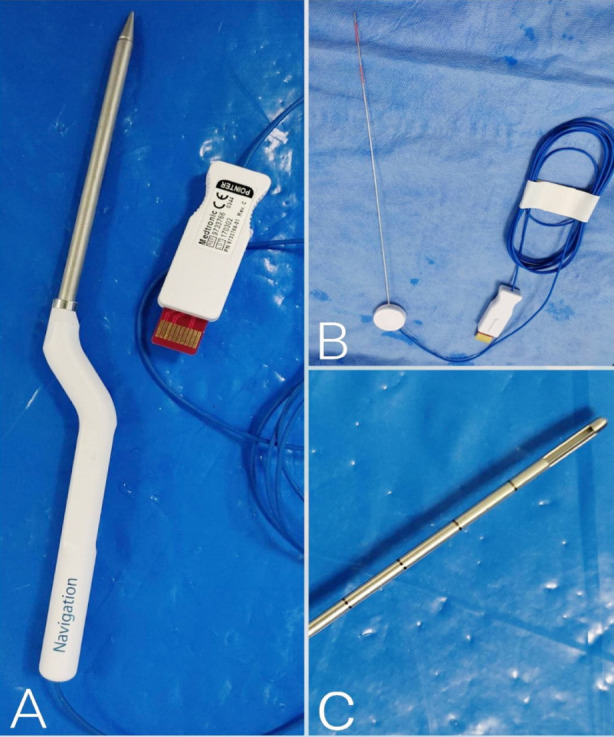
**A:** Navigation probe, **B:** EM-stylet, **C:** Biopsy cannula.

**Fig.4:**
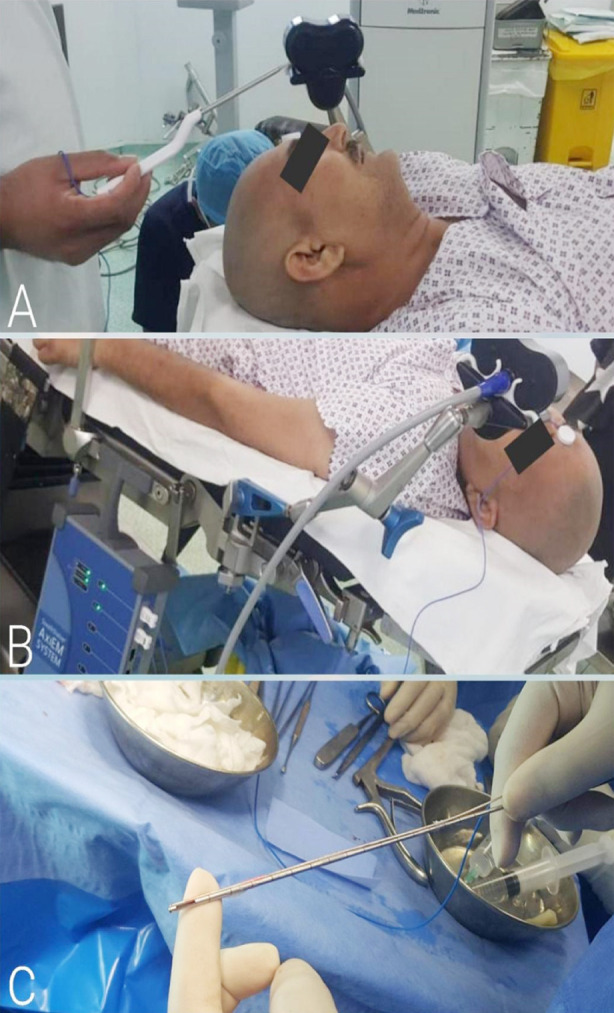
**A:** Navigation probe usage on patient and adjustment of AXIEM™ magnetic field emitter, **B:** EM-stylet placement on patient and it is attached to AXIEM™ portable system on side of operative table, **C:** EM-stylet along with biopsy cannula.

Prior trials indicate that cases of FBN are well-elicited under LA and that of FLN are better dealt by inducing GA.^4^,^13^ LA with IV sedation was the most commonly employed mode of anesthesia in our cases, i.e. in 48.9% procedures in our study, (Fig.3 and 4). Quick-Weller et al.^13^ conducted a randomized controlled trial to study the stress levels due to the type of anesthesia among patients undergoing stereotactic biopsies. The study concluded that there was no notable statistical difference between patients of GA and LA in terms of stress, except that the median values for stress were higher among the latter. Georgiopoulos et al.^7^ report a mean duration of procedure of 111.3 ± 17.2 minutes. It is considerably longer from the mean duration of procedures done at our center, 37.87 ± 9.6 minutes, implying an efficacious surgical technique and experience of surgeons in our study.

Glioblastoma WHO Grade IV was the most commonly diagnosed lesion in 44.4% biopsies, which is well-consistent with the findings of analysis by Harrisson and colleagues in which 50.66% patients had this neoplasm. An Italian research on the same topic also shows Glioblastoma WHO Grade IV to be the most commonly identified lesion in 58.01%, implying that these neoplasms are well-picked by FLN.^14^,^15^ We did not include the patients admitted on the lines of infective etiology in our study, and used FLN specifically for oncologically-suspicious intracranial lesions, similar to the inclusion criteria of another research.^14^ Though, it should be emphasized here that FLN has the ability to pick pathologies of infective or inflammatory origin, like toxoplasmosis, progressive multifocal leukoencephalopathy, multiple sclerosis and brain abscess.^2^,^7^,^10^

A recently acclaimed meta-analysis by Dhawan et al.^9^ highlights the diagnostic yield of FLN in the range of 86.6% to 100% in various clinical trials and observational studies. Kesserwan et al.^4^ also assessed the diagnostic accuracy of FLN over FBN and found similar yields of both techniques, but it is important to acknowledge that no systematic review article exists which correlates FBN and FLN with the confounding factors of size and depth of the lesions and the number of attempts made to take the sample appropriately. The diagnostic yield for FLN in our observational study was 88.9%, comparable to a Greek prospective study, i.e. 89.3%, but significantly less than a British study yielding 94.05% rate of diagnostic efficacy for FLN biopsies.^5^,^7^

The Canadian authors documented the post-procedural outcomes for FLN biopsies in their meta-analysis, for 1206 patients. The percentage mortality recorded was 2.18%, followed by the most commonly noted, 20% and 4.65% complications of asymptomatic hemorrhage and neurological deficits, respectively.^4^ We had a gross complication rate of 16.6% in our study. Asymptomatic hemorrhage was noted in 6.7% patients, followed by massive symptomatic hemorrhage in 4.4% cases.

There are only two studies in the literature that employ the AXIEM™ device for brain biopsy in the same way that we do. Giamouriadis A et al.^5^ reported 371 cases using frameless and pinless AXIEM™ system. The primary limitation of this study is that all biopsies were performed under GA, which is not appropriate for individuals with comorbidities. They also did not go into detail about the procedural details of the technique. The work done by Harrisson SE et al.^14^ is the most relevant to ours. They used the same concept of employing EM-stylet to navigate to the target and obtained 96.7% diagnostic yield, compared to 88.9% in our study. The main difference in their technique is that they fixed the biopsy needle with a trajectory guide and screw after reaching the target area. This is not a real-time navigation technique for taking a biopsy. This can increase the possibility of error if there is a small alteration in the length or direction of the needle while removing a tissue piece. Furthermore, once the needle has been positioned, it is impossible to sample in other orientations. In our technique, we navigate the needle in real time during the procedure, and any changes in position may be observed on the screen at all times. So, our technique is innovative in the sense that it offers the benefits of being frameless, pinless, and free-hand while tracking the needle position during the procedure.

### Limitations:

The retrospective methodology and small sample size of our study were primarily responsible for its shortcomings. The information collected about patients during hospitalization and follow-up was primarily derived from their medical records. As a result, bias was unavoidable when analyses were carried out using partial and inconsistent data. Biopsy cannula coupled with AXIEM™-based shunt probe (EM-stylet) for navigation may compromise target accuracy.

## CONCLUSIONS

Free-hand frameless pinless biopsy with the AXIEM™ Stealth S7 is a quick, efficient, real-time and secure neuronavigation technique for obtaining a biopsy of deep-seated lesions in the brain. In individuals who are unfit for GA, a procedure performed under LA is just as effective as one performed under GA. Patients’ head motions do not affect accuracy. The outcomes justify the use of frameless, pinless minimally invasive electromagnetic guidance over time-consuming frame-based biopsies since they are equivalent to those from frame-based procedures and can be performed as a day case. Development of new AXIEM™-based biopsy needles will further enhance accuracy as per our recommendation.

### Recommendations:

Longitudinal studies should be employed in future research. A larger sample size is necessary to strengthen the results. We recommend designing a new AXIEM™-based biopsy needle, tip of which would be compatible with AXIEM™ Emitter just like EM-stylet, to allow real time navigation and tip tracking during the procedure. Thereby, boosting maneuverability, accuracy and target precision.

### Authors’ Contribution:

**AN:** Conception and design of study, data acquisition, manuscript writing and literature review.

**AK:** Data analysis, interpretation of data, critical review of manuscript and literature review.

**HMQ:** Interpretation of results, literature review and manuscript drafting.

**MT, RB, ARB and MNH:** Data acquisition, Literature review and Manuscript editing.

**AB:** Supervision and Critical review.

All the authors have read and approved the final manuscript and are responsible and accountable for the accuracy and integrity of the work.
